# Neglected ESKAPE Pathogens in Companion Animals: Diagnostic Bias, Zoonotic Blind Spots, and Phytotherapeutic Opportunities

**DOI:** 10.3390/pathogens15090898

**Published:** 2026-08-26

**Authors:** Codruța Emilia Conțiu, Cosmina Maria Bouari, Nicodim Iosif Fiț, George Cosmin Nadăş, Sorin Răpuntean

**Affiliations:** Department of Microbiology, Immunology and Epidemiology, Faculty of Veterinary Medicine, University of Agricultural Sciences and Veterinary Medicine, 400372 Cluj-Napoca, Romania; codruta-emilia.contiu@student.usamvcluj.ro (C.E.C.); cosmina.bouari@usamvcluj.ro (C.M.B.); nfit@usamvcluj.ro (N.I.F.); sorin.rapuntean@usamvcluj.ro (S.R.)

**Keywords:** ESKAPE pathogens, companion animals, antimicrobial resistance, One Health, diagnostic bias, zoonotic transmission, phytotherapy, plant-derived antimicrobials, biofilm, GC-MS

## Abstract

The emergence of antimicrobial resistance among ESKAPE pathogens represents a major One Health challenge, yet several clinically relevant members of this group remain underrecognized in companion animals because of diagnostic limitations and research bias. This review critically evaluates current evidence on neglected ESKAPE pathogens in dogs and cats, with particular emphasis on diagnostic blind spots, antimicrobial resistance, zoonotic implications, and the potential of phytotherapeutic strategies. A structured literature search of PubMed, Scopus, and Web of Science, complemented by reports from international health organizations, was conducted to identify studies addressing pathogen occurrence, resistance mechanisms, transmission pathways, and plant-derived antimicrobial compounds. The available evidence indicates that organisms such as the *Enterobacter cloacae* complex, *Stenotrophomonas maltophilia*, *Enterococcus faecium*, and *Acinetobacter baumannii* remain substantially underinvestigated despite their clinical relevance and multidrug-resistant phenotypes. Advanced diagnostic approaches, including MALDI-TOF MS and whole-genome sequencing, together with integrated One Health surveillance, may improve their detection and epidemiological characterization. Current phytotherapeutic research demonstrates promising antibacterial, antibiofilm, and antibiotic-potentiating activities of plant-derived compounds but remains largely focused on well-characterized pathogens. Targeted investigation of neglected ESKAPE pathogens using chemically characterized plant extracts and clinically relevant veterinary isolates may support the development of complementary antimicrobial strategies and strengthen antimicrobial stewardship within the One Health framework.

## 1. Introduction

The emergence and global dissemination of multidrug-resistant (MDR) bacteria represent a major challenge for both human and veterinary medicine [1]. Among these, the so-called ESKAPE pathogens—*Enterococcus faecium*, *Staphylococcus aureus*, *Klebsiella pneumoniae*, *Acinetobacter baumannii*, *Pseudomonas aeruginosa*, and *Enterobacter* species—are of particular concern due to their ability to “escape” the effects of multiple antimicrobial agents [2]. These pathogens are traditionally associated with healthcare settings; however, their relevance extends beyond human medicine and must be considered within the framework of One Health, which recognizes the interconnectedness of human, animal, and environmental health [3].

Companion animals, particularly dogs and cats, have emerged as potential reservoirs and transmission hubs for antimicrobial-resistant bacteria. Their close and frequent contact with humans, combined with increasing access to advanced veterinary care and antimicrobial treatments, creates opportunities for the selection, persistence, and dissemination of resistant strains [4]. While well-characterized pathogens such as *E. coli*, *S. aureus*, and *K. pneumoniae* have been extensively studied in this context, other clinically relevant members of the ESKAPE group remain comparatively underinvestigated in companion animals [5].

This discrepancy is partly driven by diagnostic bias and methodological limitations. Conventional microbiological approaches, coupled with a focus on well-known pathogens, may lead to the underdetection or misidentification of less prominent bacterial species, including members of the *Enterobacter cloacae* complex and opportunistic organisms such as *Stenotrophomonas maltophilia* [6,7]. As a result, the true prevalence, ecological niches, and zoonotic potential of these organisms remain poorly defined. In many cases, the absence of evidence may reflect limitations in surveillance and diagnostics rather than a true absence.

At the same time, the therapeutic management of infections caused by ESKAPE pathogens is increasingly constrained by the rise in antimicrobial resistance mechanisms, including extended-spectrum β-lactamases (ESBLs), AmpC β-lactamases, carbapenemases, and biofilm-associated tolerance. These challenges highlight the urgent need for alternative or complementary antimicrobial strategies, particularly in veterinary settings where treatment options may be limited [3,8]. Recent experimental evidence further indicates that clinically relevant ESKAPE pathogens can rapidly evolve resistance even to antibiotics currently under development, underscoring the urgent need to identify complementary antimicrobial strategies and preserve the effectiveness of emerging therapeutic agents [9].

In this context, phytotherapy has gained attention as a potential source of bioactive compounds with antimicrobial properties. Plant-derived extracts, characterized through analytical techniques such as gas chromatography-mass spectrometry (GC-MS), contain a wide range of secondary metabolites, including terpenes, phenolic compounds, and aldehydes, that may exert antibacterial, anti-biofilm, or synergistic effects with conventional antibiotics. However, current phytotherapeutic research is largely focused on well-characterized pathogens, while neglected ESKAPE members remain insufficiently explored as potential targets [10,11].

The present review aims to address these gaps by (i) identifying underrecognized ESKAPE pathogens in companion animals, (ii) highlighting diagnostic bias and zoonotic blind spots that contribute to their underreporting, and (iii) evaluating the potential of targeted phytotherapeutic approaches against these overlooked organisms. Recent genomic and metagenomic studies have demonstrated the value of high-resolution approaches for characterizing antimicrobial resistance determinants, resistomes, and transmission patterns across diverse One Health settings, including food-borne pathogens, wildlife, and wastewater [11,12,13]. However, the application of these approaches to neglected ESKAPE pathogens at the companion-animal interface remains comparatively limited. Thus, the novelty of the present review lies not in proposing WGS or metagenomics as novel technologies per se, but in identifying this specific diagnostic and surveillance gap and integrating it with the epidemiological, zoonotic, and therapeutic challenges associated with neglected ESKAPE pathogens in dogs and cats. To the best of our knowledge, previous reviews have primarily addressed antimicrobial resistance in classical ESKAPE pathogens, broader One Health aspects of companion-animal infections, or phytotherapeutic alternatives as separate topics. In contrast, the present review integrates diagnostic limitations, epidemiological knowledge gaps, zoonotic implications, and targeted phytotherapeutic strategies within a unified One Health framework. By critically synthesizing these interconnected aspects, the review aims to identify research priorities for improving surveillance and diagnosis while directing complementary antimicrobial research toward clinically relevant but comparatively understudied veterinary pathogens.

**Figure 1:**
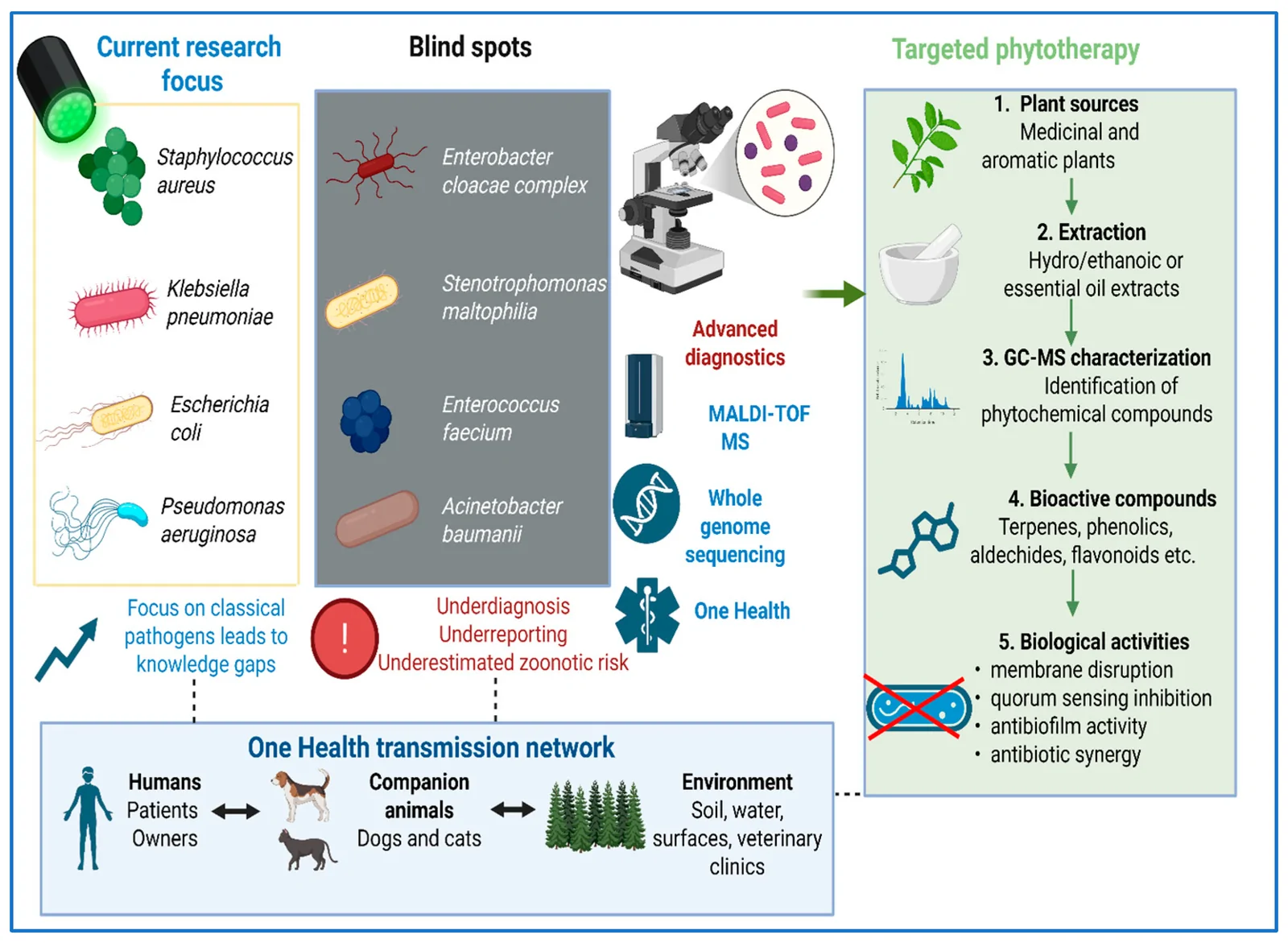
Conceptual overview of diagnostic blind spots and targeted phytotherapeutic strategies for neglected ESKAPE pathogens in companion animals within the One Health framework. Created in BioRender. Nadas, N. (2026) https://www.biorender.com/.

The conceptual framework of this review is summarized in Figure 1. The figure illustrates the central concept of this review by integrating the relationships between diagnostic bias, underrecognized ESKAPE pathogens, antimicrobial resistance, zoonotic transmission, and targeted phytotherapeutic approaches within the One Health framework. Current research predominantly focuses on classical ESKAPE pathogens (*Staphylococcus aureus*, *Klebsiella pneumoniae*, *Escherichia coli*, and *Pseudomonas aeruginosa*). In contrast, other clinically relevant organisms (*Enterobacter cloacae* complex, *Stenotrophomonas maltophilia*, *Enterococcus faecium*, and *Acinetobacter baumannii*) remain comparatively underrecognized because of diagnostic bias and limited surveillance. The proposed framework highlights the role of advanced diagnostic and surveillance approaches, including MALDI-TOF MS, whole-genome sequencing (WGS), and integrated One Health surveillance in improving pathogen detection and guiding targeted phytotherapeutic research based on phytochemical characterization (GC-MS) and evaluation of plant-derived bioactive compounds with antibacterial, antibiofilm, quorum-sensing inhibitory, and antibiotic-potentiating activities. The integration of these approaches may strengthen antimicrobial stewardship and One Health surveillance across humans, companion animals, and the environment.

## 2. Literature Search Strategy

This study was conducted as a structured narrative review aimed at identifying and critically synthesizing the available evidence on ESKAPE pathogens in companion animals, with particular emphasis on underrecognized species, antimicrobial resistance, diagnostic limitations, zoonotic relevance, and phytotherapeutic approaches. The literature search was performed using PubMed, Scopus, and Web of Science and covered publications from January 2015 to June 2026. Combinations of the following keywords were used according to the specific topic addressed: ESKAPE pathogens, companion animals, zoonotic bacteria, antimicrobial resistance, *Enterobacter cloacae* complex, *Stenotrophomonas maltophilia*, *Enterococcus faecium*, *Acinetobacter baumannii*, phytotherapy, plant extracts, and *GC-MS*.

Publications were considered relevant when they addressed one or more of the main objectives of the review, including the occurrence or clinical relevance of ESKAPE pathogens in dogs and cats, antimicrobial resistance mechanisms and phenotypes, diagnostic limitations, zoonotic or One Health implications, and the antimicrobial or antibiofilm activity of plant-derived compounds relevant to ESKAPE pathogens. Particular attention was given to studies providing information on underrecognized ESKAPE pathogens in companion animals and to studies that could help identify diagnostic, epidemiological, or therapeutic knowledge gaps. Studies dealing with well-characterized ESKAPE pathogens were also considered when they provided an appropriate comparative framework or supported the discussion of antimicrobial resistance, transmission, or phytotherapeutic mechanisms.

Titles and abstracts were screened for relevance to these objectives, followed by assessment of the full text where appropriate. Publications without a clear conceptual or scientific contribution to the topics addressed by the review were not retained for the qualitative synthesis. Because the review was intended to integrate evidence across diagnostic, epidemiological, microbiological, and phytotherapeutic domains rather than to estimate a pooled intervention effect or prevalence measure, heterogeneous types of relevant evidence were considered and synthesized qualitatively.

Emphasis was placed on recent literature, particularly studies published between 2018 and 2025, while earlier foundational studies were retained when necessary to support established concepts, methodological considerations, or antimicrobial mechanisms. Additional authoritative sources, including reports from the Food and Agriculture Organization (FAO), the World Organisation for Animal Health (WOAH), and the World Health Organization (WHO), were consulted to provide a broader One Health and antimicrobial-resistance context.

Given the structured narrative design, study selection was guided by relevance to the predefined thematic objectives. The extracted evidence was synthesized qualitatively to identify recurring patterns, inconsistencies, diagnostic and epidemiological blind spots, and priorities for future research.

## 3. ESKAPE Pathogens in Companion Animals: Current Knowledge and Biases

### 3.1. Well-Characterized Pathogens in Companion Animals

Among ESKAPE-associated bacteria, several species have been extensively studied in companion animals, particularly due to their clinical relevance, zoonotic potential, and well-documented antimicrobial resistance profiles. Notably, *S. aureus*, *K. pneumoniae*, and *E. coli* represent some of the most frequently investigated pathogens in dogs and cats [3,14,15].

*Staphylococcus aureus*, including methicillin-resistant strains (MRSA), is widely recognized for its role in skin, soft tissue, and postoperative infections in companion animals. Its zoonotic potential is well established, with numerous studies demonstrating bidirectional transmission between humans and pets, particularly in household and clinical settings. As a result, *S. aureus* has become a model organism in studies addressing antimicrobial resistance, host adaptation, and interspecies transmission [16].

Similarly, *Klebsiella pneumoniae* is a major opportunistic pathogen associated with urinary tract infections, respiratory disease, and septic conditions in companion animals. The emergence of multidrug-resistant strains, including those producing ESBLs and carbapenemases, has led to increased research attention. Its clinical importance and clear links to human infections have positioned *K. pneumoniae* as a key organism in One Health investigations [17,18].

*Escherichia coli* remains one of the most extensively studied bacterial species in both human and veterinary medicine. In companion animals, it is a common cause of urinary, gastrointestinal, and systemic infections. The widespread occurrence of antimicrobial-resistant strains, particularly ESBL-producing *E. coli*, has reinforced its role as a sentinel organism in antimicrobial resistance surveillance and zoonotic studies [19].

Despite the substantial body of literature on these pathogens, their prominence in research has contributed to an imbalance in the scientific landscape. The disproportionate focus on well-characterized organisms may obscure the presence and significance of other clinically relevant but underrecognized ESKAPE members. Consequently, current knowledge of antimicrobial resistance and zoonotic transmission in companion animals may be skewed toward a limited number of extensively studied species.

### 3.2. Neglected ESKAPE Pathogens in Companion Animals

In contrast to well-characterized pathogens, several members of the ESKAPE group remain comparatively underinvestigated in companion animals, despite their recognized clinical relevance in human medicine. These organisms are often overlooked due to diagnostic limitations, taxonomic complexity, or a research bias favoring more established pathogens. As a result, their true prevalence, antimicrobial resistance profiles, and zoonotic potential in dogs and cats remain poorly defined. The following sections highlight selected underrecognized pathogens, emphasizing current knowledge, existing gaps, and potential implications for both veterinary and public health.

#### 3.2.1. *Enterobacter cloacae* Complex

Members of the *Enterobacter cloacae* complex have been sporadically reported in infections of dogs and cats, including urinary tract infections, wound infections, and opportunistic systemic infections. However, their prevalence is likely underestimated due to limited routine screening and reporting practices in veterinary diagnostics [20,21].

The *E. cloacae* complex comprises multiple closely related species that are difficult to distinguish using conventional biochemical methods. Even with advanced techniques such as MALDI-TOF, accurate species-level identification may be inconsistent, contributing to underreporting and misclassification [7].

This group is notable for the presence of inducible AmpC β-lactamases and the increasing acquisition of ESBLs and carbapenemases. These resistance mechanisms significantly limit therapeutic options and may complicate treatment outcomes in veterinary patients [4]. Evidence for zoonotic transmission involving companion animals remains limited and largely indirect. Nevertheless, the presence of clinically relevant resistance genes suggests a potential role in the broader One Health transmission network [22].

The combination of taxonomic complexity and diagnostic bias likely contributes to the underestimation of this pathogen in companion animals. Further studies integrating molecular identification and resistance profiling are needed, particularly in the context of zoonotic risk and targeted therapeutic strategies.

#### 3.2.2. *Stenotrophomonas maltophilia*

*Stenotrophomonas maltophilia* is an environmental opportunistic pathogen occasionally isolated from companion animals, particularly in dermatological, respiratory, and wound infections [6]. Its presence in veterinary cases is increasingly recognized but remains poorly documented. Although generally identifiable using modern microbiological techniques, *S. maltophilia* may be overlooked due to its classification as a low-virulence organism or environmental contaminant [22]. This perception may reduce its clinical significance in routine diagnostics.

This species exhibits intrinsic resistance to multiple antibiotic classes, including β-lactams and aminoglycosides, and is frequently associated with multidrug-resistant phenotypes. Its ability to form biofilms further enhances persistence and treatment failure [6]. Data on zoonotic transmission involving companion animals are scarce. However, given its environmental distribution and clinical relevance in immunocompromised human patients, *S. maltophilia* may represent an underestimated interface between environmental and host-associated reservoirs [23].

The limited attention given to this organism in veterinary medicine may obscure its epidemiological significance. Greater emphasis on its detection and characterization in companion animals is needed to clarify its role in antimicrobial resistance dissemination.

#### 3.2.3. *Enterococcus faecium*

*Enterococcus faecium* is part of the normal intestinal microbiota of animals but is also implicated in opportunistic infections, including urinary tract infections, wound infections, and bacteremia in companion animals [4,20]. While generally identifiable in routine diagnostics, differentiation between commensal and pathogenic strains remains challenging. Additionally, the ecological overlap between human and animal strains complicates epidemiological interpretation [24].

*E. faecium* is a major reservoir of antimicrobial resistance, particularly in the form of vancomycin-resistant enterococci (VRE). Resistance to multiple antibiotic classes is common, contributing to its clinical significance [25]. The zoonotic role of *E. faecium* remains debated, with evidence suggesting both human-to-animal and animal-to-human transmission [20]. However, the directionality and frequency of such events are not clearly established. Despite its clinical importance, the role of companion animals as reservoirs or recipients of resistant *E. faecium* strains is insufficiently understood. Further genomic and epidemiological studies are required to clarify transmission dynamics.

#### 3.2.4. *Acinetobacter baumannii*

*Acinetobacter baumannii* has been reported in companion animals, particularly in hospital-associated infections such as wound infections and post-surgical complications [26]. However, its prevalence in veterinary settings appears lower than in human healthcare environments. Accurate identification may require advanced laboratory techniques, and its detection in veterinary practice is not always routine, potentially contributing to underrecognition [27].

This pathogen is well known for its multidrug-resistant and extensively drug-resistant (MDR/XDR) phenotypes, often associated with carbapenem resistance. These characteristics severely limit therapeutic options [28]. Although zoonotic transmission involving companion animals has been suggested, current evidence remains limited. Most studies focus on hospital-associated human strains, with less attention given to animal reservoirs [29]. The veterinary role of *A. baumannii* in antimicrobial resistance ecology is likely underestimated. Targeted investigations in companion animals are needed to determine their epidemiological and clinical significance outside human healthcare settings.

The comparative overview presented in Table 1 illustrates the marked imbalance in current research efforts across ESKAPE pathogens in companion animals and highlights the organisms that remain particularly neglected despite their clinical and One Health relevance.

Taken together, these examples demonstrate that the apparent differences among neglected ESKAPE pathogens are largely driven by common underlying factors rather than by their intrinsic biological characteristics alone. Diagnostic limitations, taxonomic complexity, uneven implementation of advanced identification methods, and the historical emphasis on well-characterized pathogens have collectively contributed to their limited recognition in companion animals. Consequently, important gaps remain regarding their epidemiology, antimicrobial resistance, zoonotic relevance, and therapeutic management, reinforcing the need for integrated surveillance and targeted research within the One Health framework.

## 4. Diagnostic Bias and Underreporting

The apparent low prevalence of certain ESKAPE pathogens in companion animals may not reflect their true epidemiological significance, but rather limitations in detection and reporting. Diagnostic bias, driven by methodological constraints and research priorities, plays a critical role in shaping the current understanding of bacterial distribution and antimicrobial resistance in veterinary settings. Conventional microbiological methods, including culture-based techniques and biochemical identification systems, remain widely used in routine veterinary diagnostics. While these approaches are cost-effective and accessible, they often lack the resolution required to identify closely related or less common bacterial species accurately. In particular, members of the *E. cloacae* complex may be misidentified or grouped at the genus level, obscuring species-specific epidemiological patterns [30,31].

The introduction of advanced technologies such as matrix-assisted laser desorption/ionization time-of-flight mass spectrometry (MALDI-TOF MS) and whole-genome sequencing has significantly improved bacterial identification and characterization. These methods allow for higher taxonomic resolution and more precise detection of antimicrobial resistance determinants [22,32]. However, their adoption in veterinary practice remains uneven, particularly in routine diagnostic laboratories, limiting their impact on surveillance of underrecognized pathogens. Taxonomic complexity further contributes to underreporting. Several clinically relevant organisms, including those within bacterial species complexes or genera with evolving classification systems, present challenges for consistent identification and reporting [4,33]. As a result, infections caused by these organisms may be underestimated or incorrectly attributed to more familiar taxa.

In addition to methodological factors, research bias toward well-known pathogens influences both study design and reporting practices. Species such as *S. aureus*, *E. coli*, and *K. pneumoniae* are frequently prioritized due to their established clinical importance and available reference frameworks [34,35]. While justified, this focus may inadvertently marginalize less-studied organisms, including *Stenotrophomonas maltophilia*, which may be dismissed as contaminants or of limited clinical relevance [6].

Collectively, these factors contribute to a systematic underestimation of certain ESKAPE pathogens in companion animals. Addressing diagnostic bias requires not only the broader implementation of advanced identification techniques but also a shift in research priorities toward a more inclusive and exploratory approach. Recognizing and correcting these blind spots is essential for a more accurate assessment of antimicrobial resistance dynamics within the One Health framework.

Furthermore, the limited research on these pathogens is also influenced by their comparatively low reported prevalence, which may reduce their perceived clinical importance and, consequently, the priority given to them in veterinary research. This creates a self-perpetuating cycle in which underrecognition leads to fewer epidemiological investigations, limiting the generation of evidence needed to improve surveillance, diagnostic protocols, and targeted therapeutic research. Breaking this cycle will require coordinated efforts to expand routine surveillance, promote the use of advanced diagnostic tools, and encourage research focused on neglected ESKAPE pathogens within the One Health framework.

## 5. Antimicrobial Resistance and Therapeutic Challenges

The increasing prevalence of AMR among ESKAPE pathogens represents a major challenge in both human and veterinary medicine. In companion animals, infections caused by MDR bacteria are becoming more frequent, particularly in clinical settings where antimicrobial exposure is common [19,21]. Resistance mechanisms such as ESBLs, AmpC β-lactamases, and carbapenemases have been increasingly reported in isolates from dogs and cats, limiting the effectiveness of commonly used antibiotics and narrowing therapeutic options [36,37].

In addition to acquired resistance, several ESKAPE pathogens exhibit intrinsic resistance traits that further complicate treatment. Organisms such as *Stenotrophomonas maltophilia* and *Enterococcus faecium* are characterized by intrinsic or frequently acquired resistance to multiple antimicrobial classes, making them particularly difficult to manage in clinical practice. Moreover, the ability of many of these pathogens to form biofilms significantly enhances their persistence in host tissues and on medical devices and abiotic surfaces. Biofilm-associated infections are notoriously resistant to antimicrobial therapy, as the extracellular matrix and altered metabolic states of bacteria reduce antibiotic penetration and efficacy [22,38,39].

In companion animals, these resistance mechanisms are often associated with chronic or recurrent infections, including otitis, dermatological infections, urinary tract infections, and post-surgical complications. Such conditions frequently require prolonged or repeated antimicrobial treatments, which may further select for resistant strains and contribute to therapeutic failure. In some cases, treatment options are limited to last-resort antibiotics, raising concerns about both animal welfare and the potential for the transmission of resistant bacteria to humans [40,41].

In veterinary medicine, therapeutic decision-making is further complicated by antimicrobial stewardship requirements, species-specific pharmacological constraints, cost considerations, and the limited availability of certain critically important antimicrobials for companion animals [1,3]. Empirical treatment may be unsuccessful when MDR organisms cause infections, while culture and susceptibility testing are not always performed before antimicrobial administration, particularly in recurrent or outpatient cases. This can delay targeted therapy and may contribute to repeated antimicrobial exposure [42,43]. Moreover, the use of last-resort or critically important antibiotics in companion animals raises ethical and public health concerns, especially when resistant bacteria have zoonotic or reverse-zoonotic potential [44].

Collectively, these challenges highlight the need for alternative or complementary therapeutic strategies. The limitations of conventional antimicrobial approaches, particularly in the context of MDR pathogens and biofilm-associated infections, provide a strong rationale for exploring novel interventions, including plant-derived compounds with antimicrobial and anti-biofilm properties.

## 6. Ecological Niches and Transmission Pathways

The ecological niches of ESKAPE pathogens in companion animals are shaped by interactions among host-associated microbiota, shared household environments, and veterinary clinical settings [4,45]. Dogs and cats may carry resistant bacteria as part of their commensal microbiota, develop opportunistic infections under conditions of disease or antimicrobial exposure, or acquire pathogens from contaminated environments [46]. Therefore, the detection of ESKAPE organisms in companion animals should not be interpreted only as an isolated clinical event, but as part of a broader ecological network involving animals, humans, and shared environments [47].

Companion-animal microbiota represent an important but still undercharacterized reservoir of antimicrobial resistance. Dogs and cats harbor complex bacterial communities on the skin, oral and nasal mucosa, urogenital tract, and gastrointestinal tract, where commensal and opportunistic bacteria may coexist with antimicrobial resistance genes [46,48]. Under normal conditions, these microbial communities may not cause disease; however, antimicrobial exposure, hospitalization, immunosuppression, wounds, surgery, or chronic inflammatory conditions can disturb this balance and favor the overgrowth or selection of resistant opportunists [45]. This is particularly relevant for organisms such as *Enterococcus faecium* and members of the *E. cloacae* complex, which may be part of intestinal or mucosal microbial communities but can also cause opportunistic infections when host or environmental conditions change [49]. Evidence from ocular microbiota studies further supports the role of companion animals as reservoirs of antimicrobial-resistant bacteria. Healthy dogs may harbor multidrug-resistant *Staphylococcus* spp. and *Pseudomonas* spp., highlighting the potential exchange of resistant strains between animals, humans, and the shared environment [50].

Veterinary clinics and household environments may also contribute to the persistence and dissemination of resistant ESKAPE pathogens. In small-animal hospitals, antimicrobial use, frequent patient turnover, invasive procedures, contaminated surfaces, and biofilm formation can create conditions that favor the maintenance of antimicrobial-resistant bacteria [47,51]. This is particularly relevant for environmentally resilient organisms such as *P. aeruginosa*, which may persist in moist surfaces, drains, plumbing systems, medical equipment, and other clinic-associated reservoirs [52]. Hospitalized animals may acquire resistant organisms during clinical care, while colonized or infected patients may contaminate cages, treatment areas, equipment, or the hands of veterinary personnel [22,53]. In the household, close contact with owners, shared living spaces, and environmental contamination may allow resistant bacteria to persist outside the clinical setting, particularly when animals have chronic infections or receive repeated antimicrobial treatment [47].

The human–animal interface represents a central component of ESKAPE pathogen ecology in companion animals. Close physical contact between pets and owners, including handling, licking, grooming, shared indoor spaces, and sometimes sleeping in the same bed, creates repeated opportunities for bacterial exchange [54]. Transmission may occur in both directions: animals may acquire resistant bacteria from humans, especially in households with prior healthcare exposure, while colonized or infected pets may contribute to human exposure through direct contact or environmental shedding [45,55]. This bidirectional exchange is particularly relevant for pathogens with known zoonotic or reverse-zoonotic potential, such as MRSA, resistant *Enterobacterales*, *E. faecium*, and *P. aeruginosa* [49,52,56].

Direct transmission primarily involves close physical contact between companion animals and humans, including bites, scratches, licking, grooming, or wound contamination. In contrast, indirect transmission may occur through contaminated household environments, veterinary equipment, shared fomites, or other environmental reservoirs that facilitate bacterial persistence and dissemination [45,47,49]. Future studies integrating One Health surveillance, whole-genome sequencing, and molecular epidemiology will be essential to distinguish these transmission pathways and better quantify their contribution to the spread of antimicrobial-resistant neglected ESKAPE pathogens [22,45,47]. Establishing transmission directionality, however, requires more than the detection of genetically related isolates in companion animals and humans. Cross-sectional identification of identical or closely related bacterial lineages may support an epidemiological link but cannot determine whether transmission occurred from animal to human, from human to animal, or through a shared environmental source. More robust inference requires longitudinal and repeated sampling of companion animals, household members, and relevant environmental sites, combined with detailed temporal and exposure data. High-resolution genomic epidemiology can complement such surveillance by assessing isolate relatedness, resistance determinants, plasmids, and other mobile genetic elements across hosts and environments [57]. As emphasized by Caddey et al. (2025) [57], longitudinal study designs integrating genomic surveillance are needed to better quantify interspecies transmission between companion animals and humans. Integrating longitudinal paired sampling with genomic and epidemiological data is therefore essential to reconstruct plausible transmission chains and distinguish bacterial sharing from evidence supporting transmission directionality [57].

Despite increasing recognition of companion animals as potential reservoirs of antimicrobial-resistant bacteria, major knowledge gaps remain regarding the ecological role of each neglected ESKAPE pathogen [45,47]. For members of the *Enterobacter cloacae* complex, it is still unclear whether dogs and cats primarily act as intestinal carriers, opportunistic hosts, or recipients of strains acquired from veterinary environments. For *Stenotrophomonas maltophilia*, the relative contribution of animal infection, environmental contamination, and shared household exposure remains poorly defined. In the case of *E. faecium*, the main uncertainty concerns the distinction between harmless colonization and clinically or epidemiologically relevant carriage of MDR or vancomycin-resistant strains. For *A. baumannii*, the role of companion animals outside hospital-associated contexts remains insufficiently understood, particularly regarding household persistence and veterinary-clinic transmission. These unresolved questions limit accurate risk assessment and highlight the need for studies combining clinical sampling, environmental surveillance, antimicrobial susceptibility testing, and genomic comparison of animal, human, and environmental isolates [22,45,51,52].

An additional limitation of the available evidence is its uneven geographical distribution. Available studies on antimicrobial-resistant bacteria in companion animals are concentrated in specific geographical settings, including several European, North American, and Asian populations [45]. Consequently, the current evidence base may not adequately represent the global epidemiology of antimicrobial resistance in companion animals. Apparent geographical differences in pathogen prevalence and resistance patterns should therefore be interpreted cautiously, as they may reflect not only true epidemiological variation but also differences in antimicrobial use, veterinary healthcare infrastructure, diagnostic capacity, and surveillance intensity [45,47]. A limited number of reports from a particular region should thus not be interpreted as evidence of a low prevalence or absence of neglected ESKAPE pathogens.

Geographical heterogeneity is also relevant to phytotherapeutic research. The chemical composition of botanical preparations may vary according to geographical origin, while harvesting, processing, and extraction conditions can further influence phytochemical profiles and biological activity [10,58,59]. Therefore, comparisons of phytotherapeutic efficacy across studies should consider both microbiological differences among circulating bacterial populations and regional variability in the plant-derived preparations being evaluated. Broader geographically representative and methodologically standardized studies are needed to determine whether reported differences reflect genuine regional variation or methodological heterogeneity.

## 7. Phytotherapeutic Alternatives: Current Evidence and Limitations

The therapeutic difficulties associated with MDR ESKAPE pathogens have renewed interest in plant-derived antimicrobial compounds as alternative or complementary strategies. In veterinary medicine, this interest is particularly relevant because recurrent infections, biofilm-associated persistence, and antimicrobial stewardship constraints may limit the long-term effectiveness of conventional treatment. Essential oils, plant extracts, and purified phytochemicals have been investigated for antibacterial, anti-biofilm, and antibiotic-modulating activities against several clinically important bacteria [10,14,40]. However, most available studies remain exploratory and are frequently focused on well-characterized pathogens rather than neglected ESKAPE members in companion animals.

### 7.1. Classes of Bioactive Compounds

Plant-derived antimicrobial activity is usually not attributable to a single molecule, but to chemically diverse mixtures of secondary metabolites [9]. Essential oils and vegetal extracts may contain terpenes, terpenoids, phenolic compounds, aldehydes, alcohols, ketones, and other small bioactive molecules, whose relative abundance depends on plant species, extraction method, geographic origin, harvesting conditions, and storage [10]. Extraction techniques and parameters can substantially influence the phytochemical profile and biological activity of plant-derived products, supporting the need for analytical characterization before antimicrobial interpretation [58,59]. In this context, approaches such as GC-MS are particularly useful for linking chemical composition with observed antibacterial, anti-biofilm, or synergistic effects.

Although this review primarily focuses on plant-derived phytotherapeutic compounds, other natural biomass sources, including algae and microalgae, are increasingly recognized as promising reservoirs of antimicrobial metabolites and represent an important direction for future One Health research [60,61].

Terpenes and terpenoids are among the most frequently investigated constituents of essential oils and plant-derived antimicrobial preparations. Their hydrophobic structure allows them to interact with bacterial membranes, potentially altering membrane permeability, disrupting cellular integrity, and impairing metabolic processes. In addition to direct antibacterial effects, essential oil constituents may interfere with biofilm formation, quorum sensing, efflux-pump activity, and resistance-modulating pathways, which is particularly relevant for MDR and biofilm-associated pathogens [62,63]. Antibacterial activity of vegetal extracts has also been reported against classical Gram-positive ESKAPE representatives such as *S. aureus*, supporting their use as benchmark organisms in phytotherapeutic screening studies [64].

Among the most extensively investigated phytochemicals, carvacrol, thymol, eugenol, cinnamaldehyde, and citral have demonstrated broad-spectrum antibacterial activity against several MDR ESKAPE pathogens. Beyond growth inhibition, these compounds have been reported to reduce biofilm biomass, interfere with quorum-sensing systems, and enhance the activity of conventional antibiotics, highlighting their potential as complementary antimicrobial agents rather than stand-alone therapeutic alternatives [65,66,67,68,69].

Phenolic compounds represent another major class of plant-derived molecules with antimicrobial relevance. This group includes simple phenolic acids, flavonoids, tannins, and phenolic monoterpenes such as thymol and carvacrol. Their antibacterial effects are often linked to membrane disruption, enzyme inhibition, oxidative stress modulation, metal ion chelation, and interference with bacterial adhesion or quorum sensing. Phenolic-rich extracts have shown activity against MDR bacteria, including ESKAPE pathogens, and may also contribute to anti-biofilm effects or synergy with conventional antibiotics [65,67,68,69].

Aldehydes are also relevant constituents of several essential oils and aromatic plant extracts. Compounds such as cinnamaldehyde and citral have been associated with antibacterial activity through interactions with bacterial membranes, disruption of cellular permeability, inhibition of enzyme activity, and interference with biofilm formation [66,67,68]. Because aldehydes are chemically reactive, their antimicrobial effects may be pronounced, but their potential cytotoxicity, volatility, and concentration-dependent activity require careful evaluation before therapeutic application. In the context of ESKAPE pathogens, aldehyde-rich extracts may therefore represent promising antimicrobial or anti-biofilm candidates. Still, their use should be supported by chemical characterization, standardized susceptibility testing, and safety assessment [66,68,69]. For clarity, the principal classes of plant-derived phytochemicals, their representative compounds, major antimicrobial mechanisms, and reported biological activities against ESKAPE pathogens are summarized in Table 2.

### 7.2. Mechanisms Relevant for ESKAPE Pathogens

One of the most relevant mechanisms of plant-derived antimicrobial compounds is the disruption of bacterial membrane integrity. Many constituents of essential oils, particularly terpenes, terpenoids, phenolics, and aldehydes, are lipophilic molecules that interact with bacterial envelopes, alter membrane permeability, disrupt ion gradients, and impair essential metabolic processes. Compounds such as carvacrol, thymol, eugenol, and cinnamaldehyde have been frequently associated with increased membrane permeability and leakage of intracellular components, leading to bacterial growth inhibition or cell death [67,68,70]. This mechanism is particularly important for ESKAPE pathogens, where reduced intracellular antibiotic accumulation, efflux activity, and cell-envelope adaptations may contribute to antimicrobial tolerance or resistance. By weakening membrane barriers, phytochemicals may not only exert direct antibacterial effects but also increase bacterial susceptibility to conventional antimicrobials [67,68,69,70].

Recent mechanistic and structure–activity evidence further indicates that the antibacterial effects of phytochemicals are strongly influenced by their molecular architecture. Carvacrol and thymol, for example, are positional isomers sharing a phenolic hydroxyl group and a hydrophobic aromatic scaffold. The phenolic hydroxyl group contributes substantially to antibacterial activity, while the hydrophobic portion of the molecule facilitates interaction with bacterial lipid membranes. Despite their structural similarity, differences in the position of the hydroxyl and other substituent groups can influence membrane interaction and biological activity. Studies of carvacrol- and thymol-derived compounds further demonstrate that the position and nature of chemical substitutions can either enhance or reduce antibacterial potency, emphasizing that relatively small structural modifications may substantially alter biological effects [71,72].

These structure–activity relationships also help explain why promising in vitro activity does not necessarily translate into equivalent biological efficacy. Increased hydrophobicity may favor interactions with bacterial membranes but can simultaneously reduce aqueous solubility and bioavailability, while volatility and limited chemical stability may further restrict therapeutic performance, as discussed by Khwaza and Aderibigbe (2025) and Latorre et al. (2025) [67,68]. Conversely, structural modification or combination with conventional antimicrobials may enhance antibacterial or antibiofilm activity. In this context, Ormeneanu et al. (2025) [73], in a pooled analysis of 216 in vitro tests, reported synergistic interactions between natural phenolic compounds and conventional antibiotics against multidrug-resistant *Klebsiella pneumoniae*. These findings further indicate that phytochemical efficacy depends not only on intrinsic molecular activity but also on compound structure, bacterial target, formulation, and experimental conditions.

Biofilm inhibition is another mechanism of particular relevance for ESKAPE pathogens, because biofilm-associated bacteria exhibit increased tolerance to antimicrobials, host immune responses, and environmental stress. Biofilm resilience is linked to extracellular matrix production, quorum-sensing-regulated virulence, reduced metabolic activity, and limited antimicrobial penetration, all of which contribute to persistence and recurrence [69,74,75,76]. Plant-derived compounds, especially essential oil constituents and phenolic molecules, may interfere with several stages of biofilm development, including initial adhesion, microcolony formation, extracellular polymeric substance production, quorum sensing, and mature biofilm stability [69,74,75]. This multi-target activity is important for pathogens such as *S. aureus*, *P. aeruginosa*, *A. baumannii*, and *E. faecium*, where biofilm formation contributes to chronic infections, device-associated persistence, and therapeutic failure [69,76]. Recent studies also suggest that some essential oils and their major components may act on both early and mature biofilms, supporting their potential role as anti-biofilm adjuvants rather than simple planktonic antibacterial agents [69,74,75].

Plant-derived compounds may also act synergistically with conventional antibiotics, an effect that is particularly relevant for MDR ESKAPE pathogens. By increasing membrane permeability, interfering with efflux systems, or weakening biofilm-associated tolerance, phytochemicals can enhance antibiotic activity and sometimes reduce the effective antibiotic concentration required for inhibition [69,73]. Recent studies have reported synergistic interactions between essential oils or phenolic compounds and antibiotics against MDR bacteria, including *S. aureus*, *A. baumannii*, and *K. pneumoniae* [69,73,77,78]. However, these effects remain strain- and method-dependent, so standardized checkerboard assays, fractional inhibitory concentration indices, time-kill studies, and clinically relevant isolate panels are needed before translation into veterinary practice [69,73].

Although these findings are promising, the reported efficacy of phytochemicals varies considerably according to the bacterial species, resistance phenotype, phytochemical composition, and experimental protocol. Consequently, standardized methodologies and studies using clinically relevant veterinary isolates are essential before these compounds can be translated into evidence-based therapeutic applications [37,58,69,78,79,80].

### 7.3. Limitations in the Literature

Despite promising results, phytotherapeutic research against ESKAPE pathogens remains limited by the predominance of simplified in vitro models [69,78,79]. Many studies evaluate antibacterial activity using planktonic reference strains under standardized laboratory conditions, which may not reflect the complexity of clinical infections in companion animals. In vivo, bacterial responses are influenced by host factors, tissue environment, antimicrobial exposure history, polymicrobial interactions, and biofilm formation [69,74,75]. As a result, activity observed in vitro does not always translate into therapeutic efficacy, particularly for chronic, recurrent, or biofilm-associated infections [69,74,75].

A second major limitation is the limited use of clinical isolates obtained from companion animals. Many phytotherapeutic studies rely on reference strains or human-derived isolates, which may not accurately represent the resistance profiles, biofilm-forming capacity, virulence traits, or host-adapted characteristics of bacteria circulating in dogs and cats. Testing plant-derived products against veterinary clinical isolates is therefore essential for evaluating their real translational potential. This approach has already been applied in veterinary microbiology beyond classical bacterial models, including studies evaluating essential oils against clinical isolates of *Prototheca* spp., and should be extended to neglected ESKAPE pathogens from companion animals [81].

A further limitation is the disproportionate focus on well-characterized or “classical” bacterial targets. In phytotherapeutic research, organisms such as *S. aureus*, *E. coli*, *K. pneumoniae*, and *P. aeruginosa* are frequently selected because they are clinically important, widely available, and supported by standardized laboratory models [58,59,65,69]. However, this focus may reproduce the same research bias observed in diagnostic and epidemiological studies. Neglected organisms such as the *Enterobacter cloacae* complex, *Stenotrophomonas maltophilia*, *Enterococcus faecium*, and veterinary-associated *Acinetobacter baumannii* are much less frequently included in phytotherapeutic screening, despite their resistance profiles, biofilm-forming capacity, and potential relevance in companion animals [6,7,26]. This imbalance limits the ability to identify plant-derived compounds with activity against the pathogens for which alternative or complementary strategies may be most needed.

### 7.4. Challenges in Clinical Translation

Despite promising in vitro antimicrobial and antibiofilm activity, the translation of plant-derived compounds into veterinary clinical practice remains constrained by several interconnected challenges. As emphasized by Vercelli et al. (2025) [82], variability in the composition of natural antimicrobial preparations represents a major obstacle to obtaining predictable efficacy and safety in companion animals. The chemical composition of essential oils and plant extracts may vary according to plant species and chemotype, geographical origin, harvesting conditions, storage, and extraction procedures [10,58,59]. Consequently, preparations obtained from the same botanical species may differ substantially in the concentrations of their major bioactive constituents and therefore in antimicrobial potency. Chemical characterization, including chromatographic profiling, and batch-to-batch quality control are thus essential prerequisites for the development of reproducible veterinary formulations [58,63,82].

A second major challenge concerns the extrapolation of experimentally active concentrations to clinically appropriate doses. As discussed by Touati et al. (2025) [69], promising antibacterial or antibiofilm effects obtained under controlled in vitro conditions may be substantially influenced in vivo by formulation, compound stability, bioavailability, and the biological environment. MIC or antibiofilm concentrations therefore cannot be directly translated into therapeutic doses in dogs and cats. Absorption, distribution, metabolism, route of administration, target-tissue concentrations, and interactions among individual constituents may all modify biological activity and should be considered during preclinical development [63,82]. These limitations reinforce the need for pharmacokinetic and pharmacodynamic studies before antimicrobial activity observed in vitro can be interpreted as evidence of therapeutic potential [63,69,82].

Safety represents an equally important translational barrier. Sivamaruthi et al. (2024) [63] emphasized that, despite the growing use of essential oils and other plant-derived preparations in domestic animals, available toxicological evidence remains limited and the effects of dose and repeated exposure require careful consideration. Safety cannot be inferred solely from the natural origin of a compound, and concentrations displaying antimicrobial activity may not necessarily provide an acceptable therapeutic margin in vivo. Potential local irritation, systemic toxicity, interactions with conventional medications, and species-specific differences in metabolism should therefore be evaluated before clinical application [63,82]. This consideration is particularly relevant in companion-animal medicine, where evidence obtained in other animal species or experimental models cannot automatically be extrapolated to dogs and cats [63,82].

Regulatory and product-development requirements constitute an additional barrier. Vercelli et al. (2025) [82] highlighted the lack of harmonized regulatory frameworks for natural antimicrobial products and the difficulties created by variability in composition, quality, and therapeutic claims. Depending on the intended use and jurisdiction, plant-derived preparations may fall under different regulatory categories, with corresponding differences in requirements for demonstrating quality, safety, and efficacy [82]. The intrinsically complex composition of botanical preparations further complicates conventional pharmaceutical standardization and regulatory assessment [63,82].

Taken together, these limitations indicate that promising in vitro activity should be considered an initial step in the translational pathway rather than evidence of clinical efficacy. Progress toward evidence-based phytotherapeutic applications in companion animals will require chemically characterized and standardized preparations, reproducible manufacturing and quality-control procedures, species-specific toxicological and pharmacokinetic studies, rational dose determination, appropriate formulations, and ultimately controlled clinical trials with clearly defined therapeutic and safety endpoints [63,69,82]. Until such evidence becomes available, plant-derived antimicrobials should be regarded primarily as promising complementary candidates rather than established substitutes for conventional antimicrobial therapy [63,69,82].

## 8. Bridging the Gap: Targeted Phytotherapy for Neglected Pathogens

The evidence reviewed above suggests that phytotherapeutic research should move beyond broad screening against classical bacterial models and toward targeted testing against neglected ESKAPE pathogens from companion animals. Rather than evaluating plant-derived products randomly, future studies should select bacterial targets based on diagnostic blind spots, resistance profiles, ecological relevance, and therapeutic need, especially where current veterinary data remain fragmented. This approach is particularly important for members of the *Enterobacter cloacae* complex, which may be underreported because of taxonomic complexity and diagnostic limitations [7], and for *Stenotrophomonas maltophilia*, an opportunistic pathogen that remains poorly characterized in companion animals despite intrinsic resistance and biofilm-forming capacity [6]. Similar gaps persist for *Enterococcus faecium*, where the distinction between commensal carriage and clinically relevant MDR or vancomycin-resistant strains remains difficult [24], and for veterinary-associated *Acinetobacter baumannii*, whose role in companion animals outside hospital-associated contexts is still insufficiently defined [26].

The growing discrepancy between the increasing prevalence of AMR and the limited development of novel antibacterial agents has renewed interest in phytotherapeutic approaches as complementary tools for managing infections caused by ESKAPE pathogens. Recent reviews highlight plant-derived compounds as promising sources of antibacterial, antibiofilm, and resistance-modifying agents that target multidrug-resistant bacteria through mechanisms distinct from those of conventional antibiotics [79,83]. From a One Health perspective, future antimicrobial discovery efforts should therefore prioritize clinically relevant resistant pathogens and integrate animal, human, and environmental reservoirs into antimicrobial research frameworks [45,47,83].

A critical step toward addressing this gap is the integration of phytochemical characterization with microbiological testing. Modern analytical techniques, particularly gas chromatography–mass spectrometry (GC-MS), enable the detailed identification of volatile and semi-volatile constituents present in essential oils and plant extracts, facilitating the correlation of chemical composition with observed biological activity [58]. This approach is increasingly recognized as essential because the antimicrobial efficacy of plant-derived products often results from synergistic interactions among multiple compounds rather than from a single dominant constituent [64]. Recent investigations have demonstrated that phytochemicals such as carvacrol, thymol, eugenol, cinnamaldehyde, and citral can exert antibacterial effects through membrane disruption, interference with quorum sensing, inhibition of biofilm formation, and modulation of bacterial stress responses [62,74,75]. Importantly, the activity of these compounds varies considerably according to both extract composition and bacterial target, highlighting the need for chemically characterized preparations when evaluating antimicrobial potential [62,69]. Consequently, combining GC–MS profiling with susceptibility testing against neglected ESKAPE pathogens may provide a more rational framework for identifying promising phytotherapeutic candidates and elucidating their mechanisms of action, particularly in the context of multidrug-resistant infections affecting companion animals [58,69].

Future studies should prioritize contemporary multidrug-resistant isolates recovered from companion-animal infections rather than relying predominantly on laboratory reference strains. Integrating such clinically relevant isolates with standardized susceptibility testing and phytochemical characterization would provide a more realistic framework for identifying plant-derived compounds with genuine translational potential [21,22,40,51,81,84].

Beyond bacterial growth inhibition, future phytotherapeutic investigations should incorporate endpoints that better reflect the biological characteristics of neglected ESKAPE pathogens. Biofilm formation is a major determinant of persistence, antimicrobial tolerance, and therapeutic failure in organisms such as *Stenotrophomonas maltophilia*, *Enterococcus faecium*, *Acinetobacter baumannii*, and members of the *Enterobacter cloacae* complex [6,24,26,69,74,76]. Within biofilms, bacteria exhibit altered metabolic activity, enhanced protection from host immune responses, and reduced susceptibility to antimicrobial agents, making eradication substantially more difficult than in planktonic cultures [69,74,75,85]. Consequently, the identification of plant-derived compounds that interfere with adhesion, quorum sensing, extracellular matrix production, or mature biofilm stability may offer greater clinical value than the discovery of compounds that act solely through bactericidal mechanisms [69,74,75,85].

In parallel, increasing evidence suggests that phytochemicals can enhance the activity of conventional antibiotics through membrane permeabilization, efflux-pump inhibition, and disruption of resistance-associated adaptive responses [67,68,69,73]. Synergistic interactions between essential oils, phenolic compounds, and conventional antimicrobials have already been reported against multidrug-resistant ESKAPE pathogens, supporting their potential role as adjunctive rather than replacement therapies [69,73,77,86]. Prioritizing anti-biofilm and antibiotic-potentiating activities during the screening of GC–MS-characterized plant extracts against neglected veterinary pathogens may therefore provide a more clinically relevant pathway for phytotherapeutic development within the One Health framework [54,69,74,86].

## 9. Future Perspectives: Toward Targeted Phytotherapeutic Strategies for Neglected ESKAPE Pathogens

Although considerable progress has been made in understanding antimicrobial resistance among ESKAPE pathogens, significant knowledge gaps remain regarding neglected species circulating in companion animals. Future research should move beyond descriptive epidemiological studies and adopt integrated approaches that combine microbiology, molecular epidemiology, phytochemistry, and One Health surveillance [3,45,78,83,87]. Such strategies are essential for accurately defining the epidemiological role of underrecognized pathogens, clarifying transmission dynamics at the human–animal interface, and identifying novel therapeutic opportunities [45,78,83,87].

A major priority is the standardization of phytotherapeutic research. Considerable methodological heterogeneity currently exists regarding plant material selection, extraction procedures, phytochemical characterization, and antimicrobial susceptibility testing, making direct comparison among studies difficult [69,84,88,89,90]. The implementation of harmonized protocols, together with comprehensive chemical profiling using analytical techniques such as GC–MS, would substantially improve reproducibility and facilitate the identification of bioactive compounds with genuine antimicrobial potential. Standardized evaluation of antibacterial, antibiofilm, and antibiotic-potentiating activities should also become routine in future investigations [69,73,74,84,86,90,91].

Future studies should also prioritize clinically relevant veterinary isolates rather than relying predominantly on laboratory reference strains. The inclusion of multidrug-resistant isolates obtained from companion animals would provide a more realistic assessment of phytotherapeutic efficacy and better reflect the diversity of resistance mechanisms encountered in veterinary practice [21,22,40,51,81,92,93]. At the same time, integrating whole-genome sequencing and complementary metagenomic approaches with phenotypic susceptibility testing and phytochemical analyses would enable correlations between resistance determinants, virulence factors, and responsiveness to plant-derived compounds, providing a stronger mechanistic basis for antimicrobial discovery and One Health surveillance [11,12,13,38,48,87].

Recent large-scale genomic studies further illustrate the value of genomic surveillance for detecting emerging antimicrobial resistance patterns and tracking their geographical dissemination. Global genomic analysis of *Enterobacter hormaechei*, a member of the *Enterobacter cloacae* complex, revealed a high prevalence and broad distribution of clinically important carbapenem resistance genes, including *bla*NDM-1, *bla*IMP-4, *bla*KPC-3, *bla*VIM-1, *bla*NDM-5, *bla*OXA-48, and *bla*KPC-2 [94]. In parallel, recent global genomic surveillance studies have identified increasing azithromycin resistance, together with resistance or decreased susceptibility to ciprofloxacin and third- and fourth-generation cephalosporins [95,96]. Collectively, these findings demonstrate the capacity of genomic approaches to identify emerging resistance determinants and temporal and geographical resistance trends, supporting the expansion of comparable surveillance strategies to neglected ESKAPE pathogens within a One Health framework.

Finally, translating promising in vitro findings into veterinary applications will require carefully designed in vivo studies addressing pharmacokinetics, safety, formulation, and clinical efficacy in companion animals [63,69,82]. Rather than replacing conventional antimicrobials, phytotherapeutic compounds are more likely to serve as complementary strategies that enhance antibiotic activity, reduce biofilm-associated persistence, and support antimicrobial stewardship [69,73,86,97,98,99,100]. Within a One Health framework, such integrated research may contribute to the development of innovative therapeutic approaches while improving surveillance and control of neglected ESKAPE pathogens across animal, human, and environmental reservoirs [3,45,47,78,83].

## 10. Conclusions

Companion animals are increasingly recognized as important components of the One Health network of antimicrobial resistance, acting not only as recipients of multidrug-resistant bacteria but also as potential reservoirs involved in the circulation of clinically relevant ESKAPE pathogens. However, current knowledge remains disproportionately focused on a limited number of well-characterized organisms, particularly *Staphylococcus aureus*, *Klebsiella pneumoniae*, and *Escherichia coli*. In contrast, pathogens such as the *Enterobacter cloacae* complex, *Stenotrophomonas maltophilia*, *Enterococcus faecium*, and *Acinetobacter baumannii* remain comparatively underinvestigated in companion animals, despite their recognized importance in human medicine. This imbalance is likely driven by diagnostic limitations, taxonomic complexity, and research priorities that favor more familiar bacterial species.

The growing prevalence of antimicrobial resistance, together with the intrinsic resistance and biofilm-forming capacity of many neglected ESKAPE pathogens, highlights the limitations of conventional therapeutic approaches in veterinary medicine. Plant-derived antimicrobial compounds represent promising complementary strategies because of their ability to target multiple bacterial processes, including membrane integrity, quorum sensing, biofilm formation, and antibiotic susceptibility. Nevertheless, current phytotherapeutic evidence remains fragmented and largely limited to in vitro studies conducted on reference strains or a small number of well-studied pathogens. Future research should therefore prioritize neglected ESKAPE organisms isolated from companion animals and integrate advanced diagnostics, genomic surveillance, phytochemical characterization, and clinically relevant veterinary isolates to improve their epidemiological characterization and evaluate targeted phytotherapeutic strategies. Such integrated research may support the development of evidence-based complementary antimicrobial approaches while strengthening antimicrobial resistance surveillance in companion animals.

## Figures and Tables

**Table 1:** Comparative overview of ESKAPE pathogens in companion animals, highlighting current research intensity, zoonotic evidence, antimicrobial resistance, phytotherapeutic investigation, and major knowledge gaps.

Pathogen	Research Intensity	Zoonotic Evidence	AMR Level	Phytotherapeutic Exploration	Critical Remarks
*Enterobacter cloacae complex*	Limited–moderate	Limited, indirect	High (AmpC, ESBL, carbapenemases)	Very low	Taxonomic complexity → likely underreported; rarely targeted in phytotherapy studies
*Stenotrophomonas maltophilia*	Limited	Very limited	Intrinsically high (MDR)	Limited	Environmental opportunist; largely overlooked in zoonotic frameworks
*Enterococcus faecium*	Moderate	Ambiguous (unclear directionality)	Very high (VRE)	Limited	Better studied in humans than animals; limited targeted phytotherapy data
*Acinetobacter baumannii*	Moderate	Limited	Very high (MDR/XDR)	Limited–moderate	Dominated by hospital-centered literature; veterinary ecology underexplored
*Klebsiella pneumoniae*	Extensive	Moderate–well documented	Very high (ESBL, KPC, NDM)	Moderate	Well studied; useful as a benchmark/control organism
*Escherichia coli*	Extensive	Well documented	High (ESBL)	Extensive	Overrepresented in both AMR and phytotherapy studies
*Staphylococcus aureus*	Extensive	Well documented (MRSA)	High	Extensive	Classical model organism; strong literature bias

**Table 2:** Major classes of plant-derived phytochemicals relevant to neglected ESKAPE pathogens and their principal antimicrobial properties.

Phytochemical Class	Representative Compounds	Principal Antimicrobial Mechanisms	Reported Biological Activities	Representative ESKAPE Pathogens
Terpenes and terpenoids	Carvacrol, thymol	Membrane disruption, increased permeability, quorum-sensing interference	Antibacterial, antibiofilm, antibiotic potentiation	*S. aureus*, *A. baumannii*, *K. pneumoniae*
Phenolic compounds	Eugenol, flavonoids, tannins	Membrane damage, enzyme inhibition, oxidative stress modulation	Antibacterial, antibiofilm, synergistic effects	*S. aureus*, *E. faecium*, *P. aeruginosa*
Aldehydes	Cinnamaldehyde, citral	Membrane disruption, inhibition of biofilm formation	Antibacterial, antibiofilm	MDR Gram-negative ESKAPE pathogens

Compiled from references [55,56,57,58,59,60,61,62,63,64,65,66,67,68,69,70,71,72,73,74,75].

## Data Availability

No new data were created or analyzed in this study. Data sharing is not applicable to this article.
